# Lysyl Oxidases: Orchestrators of Cellular Behavior and ECM Remodeling and Homeostasis

**DOI:** 10.3390/ijms231911378

**Published:** 2022-09-27

**Authors:** Shelly Zaffryar-Eilot, Peleg Hasson

**Affiliations:** Department of Genetics and Developmental Biology, The Rappaport Faculty of Medicine and Research Institute, Technion–Israel Institute of Technology, Haifa 3525422, Israel

**Keywords:** lysyl oxidase, extracellular matrix, epithelial to mesenchymal transition, transcriptional regulation, intracellular activity

## Abstract

Lysyl oxidases have long been considered key secreted extracellular matrix modifying enzymes. As such, their activity has been associated with the crosslinking of collagens and elastin, and as a result, they have been linked to multiple developmental and pathological processes. However, numerous lines of evidence also demonstrated that members of this enzyme family are localized and are active within the cytoplasm or cell nuclei, where they regulate and participate in distinct cellular events. In this review, we focus on a few of these events and highlight the intracellular role these enzymes play. Close examination of these events, suggest that the intracellular activities of lysyl oxidases is mostly observed in processes where concomitant changes in the extracellular matrix takes place. Here, we suggest that the LOX family members act in the relay between changes in the cells’ environment and the intracellular processes that promote them or that follow.

## 1. Introduction

**Figure 1:**
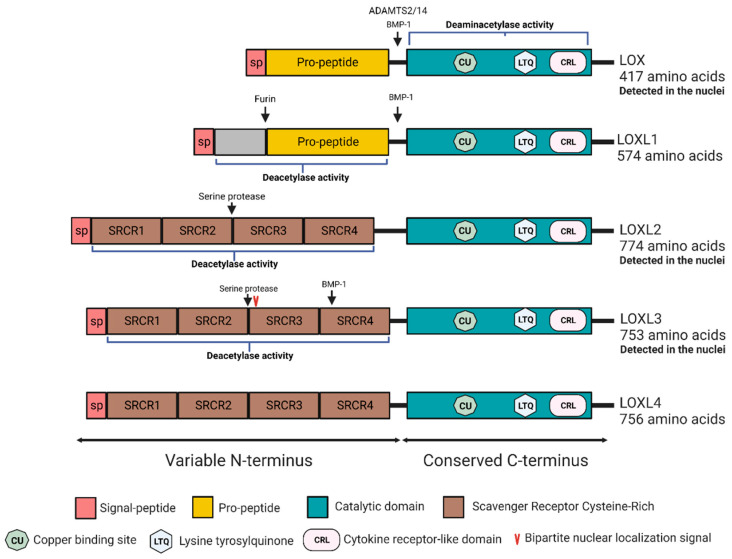
Schematic domain structure and homology of the lysyl oxidase family members. All five members of the lysyl oxidase family share a highly conserved C-terminal domain comprising the catalytic domain with a copper-binding motif, cytokine receptor-like domain, and lysyl tyrosyl quinone (LTQ) cofactor required for protein conformation and catalytic activity, respectively. The N-terminal regions of LOX proteins are more variable: LOX and LOXL1 contain a propeptide sequence, while LOXL2, LOXL3, and LOXL4 contains four scavenger receptor cysteine-rich (SRCR) domains. LOXL3 contains bipartite nuclear localization signal (red arrowhead). Black arrow represent putative cleavage sites.

Lysyl oxidases (LOX) are a family of highly conserved copper-dependent enzymes comprising five members: lysyl oxidase (LOX) and four lysyl oxidase-like enzymes (LOXL1-4) (Figure 1). They were first discovered as a secreted extracellular matrix (ECM) modifying enzymes essential for crosslinking and stabilizing collagen and elastin fibers [1].

Lysyl oxidases catalyze the oxidative deamination of lysine and hydroxylysine residues of collagens and elastin to generate the highly reactive corresponding allysine. The allysines can then spontaneously condense with other lysines and hydroxylysines residues to form the crosslinked mature collagens and elastin [1,2,3]. β-aminopropionitrile (βAPN), a pan irreversible inhibitor of LOX(L) proteins [4] was first discovered in the context of Lathyrsm and was therefore known as a lathyritic agent [5]. βAPN was first isolated from the sweet pea, *L. odouratus*, [6], and its administration resulted in aortic rupture, severe skeletal abnormalities, and connective tissue fragility [5]. 

All members of the enzyme family share a highly conserved catalytic domain at their C-termini. This region contains the lysine tyrosylquinone (LTQ) cofactor and a copper binding motif, which are both essential for their catalytic activity [7]. The N-termini are more divergent; LOXL2-4 share a common structure at their N-terminus encompassing four tandem repeats of scavenger-receptor-cysteine-rich (SRCR) domains. SRCR domains assume to entail protein–protein interaction sites. In contrast to LOXL2-4, LOX and LOXL1 lack the SRCR domains but contain a pro-peptide (PP) sequence at the N-terminus, which is cleaved at the ECM and is critical for their activation. LOX and LOXL1 are both secreted to the ECM as inactive proenzymes, where they undergo proteolytic cleavage by Bone morphogenetic protein-1 (BMP-1, also known as procollagen C-proteinase) or ADAMTS proteins inducing their activation [8,9] (Figure 1). Distinct activities have been associated with various domains of the protein, further reinforcing the observations of the multitude roles played by the members of this enzyme family.

**Figure 2:**
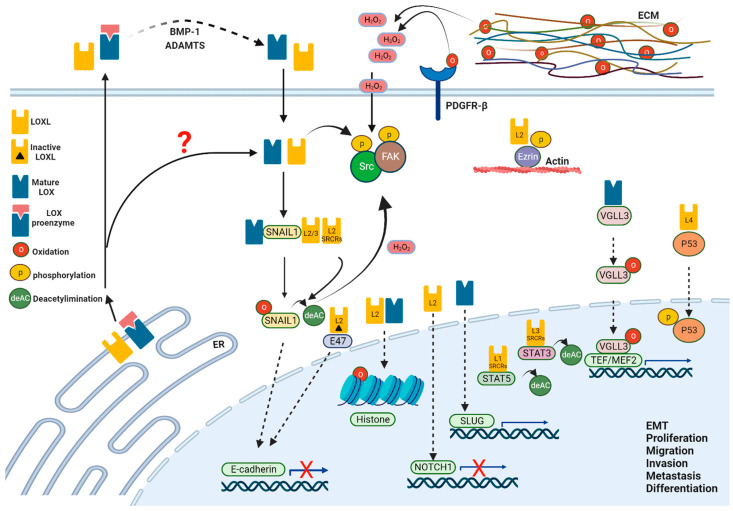
Summary of various extracellular and intracellular LOX family activities. LOX and LOXL secreted to the extracellular matrix. LOX secreted in its proenzyme form undergoes proteolytic cleavage mostly by BMP-1 to generate the mature active enzyme. The putative mechanism for LOX and LOXL intracellular processing is unknown yet, but it is possible they are secreted from the ER and undergo intracellular proteolytic cleavage and post-translational modifications to generate intracellular mature active enzymes. LOX and LOXL oxidize and deacetylate various proteins, thus contributing to their stabilization.

LOX family members have been associated with the development and regeneration of numerous tissues and have been implicated in a variety of diseases affecting multiple organs [10,11,12,13,14,15]. It has now been well-documented that members of this enzyme family are major players in ECM remodeling; however, growing evidence implicates them also as having intracellular cell-autonomous activities (Figure 2). These findings may enable LOX enzymes to play key roles in the communication between the cells’ environment and intracellular processes to pass information between the extracellular space and the cells’ interior, enabling remodeling of the ECM or altering cellular behavior as a feedback mechanism. In this review, we focus on a variety of LOX-dependent intracellular activities that enable lysyl oxidases to act as such unique sensing enzymes.

## 2. Lysyl Oxidases: Multi-Faceted Regulators—The Case of EMT

The involvement of the lysyl oxidases in epithelial-to-mesenchymal transition (EMT), a complex process by which epithelial cells lose their cell–cell adhesions and acquire migratory properties, has been well-documented and, as such, serves as an ideal model for demonstrating the multiple roles the LOX proteins play [16,17,18,19]. EMT is a crucial process in embryonic development [20] and in tumor progression, enabling epithelial tumor cells to metastasize, to escape from the primary tumor, to migrate, and to invade to a distant tissue [21,22,23,24,25]. The processes of cell detachment and subsequent migration involve multiple intracellular changes, including gene expression changes and cytoskeletal rearrangements, and changes in the cells’ environment. Interestingly, multiple lines of evidence directly link lysyl oxidases not only to ECM modifications but also to intracellular changes occurring during EMT.

A key event in EMT is the transcriptional down-regulation of the adherens junction protein E-Cadherin (E-Cad) mediated by the transcription factor Snail1 (Snai1) [26]. Lysyl oxidases were initially identified as key enzymes controlling this process by physically interacting with and directly oxidizing Snail1, thus stabilizing it in the perinuclear envelope, resulting in the down regulation of E-Cad (Figure 2) [16,17]. Moreover, LOXL2 was shown to collaborate with the bHLH transcription factor E47 in the perinuclear compartment to regulate E-Cad repression (Figure 2) [27]. Notably, E-Cad is not the only transcriptional target of lysyl oxidases during EMT. Indeed, LOX is also recruited to the promoter of Slug, a transcription factor of the Snail family (also known as Snai2) to induce EMT (Figure 2), at least in part via the transcriptional regulation of Tissue inhibitor of metalloproteinase 4 (TIMP4), which in turn regulates the proteolytic activity of Matrix metalloproteinases (MMPs) [28].

While the above demonstrations highlight several modes of LOXs induction of EMT, primarily via regulation of E-Cad, other lines of evidence suggest alternative modes also exist. Indeed, up-regulation of LOXL2 promotes EMT and gastric cancer metastasis through the Src/FAK pathway [29]. Interestingly, the activation of the Src/FAK pathway is mediated, at least in part, via hydrogen peroxide, a by-product of lysyl oxidases’ enzymatic activity (Figure 2) [29,30]. In breast carcinoma cells, enzymatically inactive LOXL2 was found to induce EMT via alternate means. Here, LOXL2 was shown to be involved in cell polarity and tight junction complex organization by downregulation of Lethal giant larvae (Lgl2) and claudin1 genes, respectively, thus acting in an independent mechanism to the Snail and E-Cad pathway [31]. Altogether, lysyl oxidases induce intracellular changes to promote EMT in multiple mechanisms, enzymatic and non-enzymatic, directly or indirectly, irrespective of their activities in the ECM and their coordinated functions enable a smooth transition.

## 3. Lysyl Oxidases Regulate Cytoskeletal Assembly

Recent work has implicated members of the LOX family in cytoskeletal organization, at least to some extent, in a catalytically independent-manner. LOXL2 was shown to promote esophageal squamous cell carcinoma (ESCC) migration and invasion. In this process, LOXL2 and an alternatively spliced enzymatically inactive variant L2D13, physically interact with Ezrin (an intracellular actin-binding protein) to enhance Ezrin phosphorylation, promote actin cytoskeleton reorganization, and subsequently filopodia formation (Figure 2) [32].

In agreement with these findings, we recently found that LOX is also involved in cytoskeletal organization in an enzyme-independent manner in smooth muscle cells (SMCs). Following LOX knockdown, SMCs exhibit disruption of actin and microtubule organization. In addition, multiple actin-binding protein were found spread across the cytoplasm but did not align with the actin fibers. Notably, this regulation of actin cytoskeleton is not only cell autonomous but also ECM-independent and is carried out via regulation of myosin light chain phosphorylation and the Rho-associated kinases’ ROCK1 and ROCK2 expression [33].

## 4. LOX-Dependent Transcriptional Regulation

Lysyl oxidases have been involved transcription regulation, at least in part, via modulating transcription factor activity and subcellular localization. As mentioned above, a physical interaction between members of the lysyl oxidase family with Snail1 to regulate its activity has been demonstrated. Interestingly, such functions are not anecdotal as LOXL4 directly bind and stabilizes TP53 (p53) in the cytoplasm, resulting in p53 phosphorylation and subsequent accumulation in the nuclei (Figure 2) [34]. In addition, we have recently identified a novel, autonomous role for Lox in regulating muscle progenitor cells’ differentiation. This activity was mediated via the oxidation of the transcriptional co-activator Vestigial Like 3 (Vgll3), a partner of myocyte enhancer factor 2 (Mef2) proteins and of transcriptional enhancer factor (TEF) (also known as TEAD) proteins, key transcriptional regulators crucial in multiple tissues [35,36]. Here, oxidation regulated the dynamics of Vgll3 nuclear translocation to promote expression of genes associated with myogenic differentiation (Figure 2) [10].

In order to facilitate their intracellular roles, lysyl oxidases may translocate into the cell or into the nuclear compartment, although the exact mechanism underlying their intracellular localization is currently unknown. Many indications have demonstrated the presence of lysyl oxidases in the nuclei or cytoplasm of various cells, including epithelial cells, neuroglial cells, myoblasts, and satellite cells [10,16,31,37,38]. One such example is LOX-V2, a novel variant of LOX lacking the N-terminus, including the propeptide region, and as such remains intracellularly. LOX-V2 was found to colocalize with the promyelocytic leukemia-nuclear bodies (PML-NBs) in nuclei, raising the possibility that it can regulate various cellular activities [39]. While the above LOX-V2 isoform does not contain a signal peptide, other LOX family members that contain such export signals are also found intracellularly. Accordingly, cytoplasmic and perinuclear LOXL2 was found in human laryngeal squamous cell carcinomas (LSCC) and human breast carcinoma cells, and this expression pattern correlated with malignant progression and increased metastasis [31,40]. 

By combining immunofluorescent analysis, nuclear isolation followed by LOX activity assays, Kagan and colleagues demonstrated the presence of active LOX within the nuclei of vascular smooth muscle cells (VSMCs), and the presence of mature LOX at the intra-nuclear and peri-nuclear compartment of fibroblasts [41]. In another study, transforming growth factor-β (TGFβ) was shown to directly increase nuclear LOX protein expression in interstitial fibroblasts isolated from O_2_-injured (85% O_2_) mouse pup lung [42]. Additional studies demonstrating nuclear activity of LOX focused on its’ tumor-suppressor properties in the ras-transformed cells, RS485 (NIH 3T3 transformed by c-H-ras), cells which normally express low LOX levels [43,44,45,46]. Interestingly, the over-expression of the 50-kDa proenzyme led to the inhibition of cell growth in soft agar [44,45] and reduced tight chromatin packing state. In contrast, LOX knockdown in the parental NIH 3T3 non-transformed cells enhanced chromatin condensation, demonstrating LOX roles in chromatin remodeling [46].

Overall, the above demonstrations indicate that LOX can localize to the nucleus, where it plays a functional role in gene regulation, also via chromatin organization (Figure 2). Indeed, in vitro analysis demonstrated that LOX interacts with and oxidizes Histone H1 [47], an interaction that was identified due to the similarities in sequences in this histone to those demonstrated for LOX interaction site with Tropoelastin (AKA sequence (AKAAAKAAAKA) [48,49]). Interestingly, the histone H2 protein also shares a resemblance to the AKA sequence although treatment with the AKA blocking peptide only partially abolished this interaction, raising the possibility that the interaction between LOX and Histone H2 is also mediated via an additional domain. In support of these observations were results obtained using a carboxy-terminal deleted version of LOX (Δ-LOX) that only partially hindered these interactions [48], suggesting that LOX interacts with these histone proteins via more than one domain. Interestingly, these changes in chromatin condensation were accompanied by changes in cell–cell adhesion properties through the cadherin/catenin pathway [50,51]. In addition to the histone association, LOX was also found to interact with several nuclear proteins. One of them, p66β, a protein of the nucleosome remodeling repressor complex Mi2/NuRD, is involved in E-Cad gene repression and plays an essential role in the LOX-dependent histone oxidation [52,53].

Notably, in addition to LOX, other members of the family were also found to entail nuclear activities and to regulate transcription. LOXL2 directly interacts with the NOTCH1 promoter to suppress NOTCH1 expression (Figure 2) in squamous cell carcinoma (SCC), contributing to tissue homeostasis [54]. LOXL3 modulates STAT3 activity via deacetylating/deacetyliminating the latter (Figure 2), thus regulating STAT3 activity and inducing immune cell proliferation, differentiation, and inflammatory response. Interestingly, this interaction was independent of the catalytic activity of LOXL3, and moreover, LOXL3 SRCR repeats alone could induce this chemical reaction even without the catalytic domain. In addition, the N-terminal domain of LOXL1 and the LOXL2-SRCR domain resulted in STAT5 and Snail deacetylation, respectively, suggesting this could be a more common activity and mode of regulation mediated by the LOXL proteins [38]. 

## 5. Perspectives

Accumulating evidence demonstrates that, on top of their extracellular activities, lysyl oxidases also have intracellular activities. Notably, many of these intracellular activities are associated with the cells’ response to changes in the ECM, where ongoing crosstalk between the intra- and extracellular environments are critical. Below, we highlight a few examples where such an orchestration between intracellular and extracellular events is essential.

Increasing evidence indicates that ECM organization and its mechanical properties significantly modulate EMT, ensuring that matrix organization can facilitate the changes in the cells, including the acquiring of a migratory and invasive properties. Reciprocally, cells undergoing EMT induce changes in ECM organization, leading to an adapted ECM that can accommodate the differences in the cells’ properties [55]. Thus, the mechanisms described above might enable the adaptation of the intracellular responses to the required ECM alterations.

Consistent with this, we have recently demonstrated that LOX regulates satellite cell differentiation by regulating the dynamics of the cytoplasmic-to-nuclear shuttling of Vgll3, a transcription factor participating in the myogenic differentiation program. Thus, within the skeletal muscle connective tissue, LOX acts at the cells’ exterior to organize and remodel the ECM in parallel to its intracellular role within myogenic progenitor cells to promote their differentiation state. This mechanism of crosstalk may ensure that differentiation of myogenic progenitors will occur only when the surrounding matrix is capable of maintaining the growing muscle fiber [10]. Likewise, we suggest that LOX regulation of cytoskeletal organization in vascular smooth muscles (Aviram in preparation) is a mechanism that ensures that the cells can appropriately contract and exert force in response to the alterations in the ECM, which are too LOX-dependent [14,56,57].

Altogether, lysyl oxidases have multiple functions; they act at the extracellular space, where they are key regulators of ECM organization (e.g., collagen and elastin crosslinking) but they also function intracellularly, in the cytoplasm and nucleus, by interacting with and oxidizing transcription factors, histones, and other intracellular proteins. Along these lines, Salvador et al. [58] showed an exclusive intracellular role for LoxL2 in promoting lung metastasis of breast cancer cells through the SNAIL pathway without any effect on ECM organization or rigidity. It should be noted that studies showing additional functions of lysyl oxidases at the cells’ exterior, in which they interact with and oxidize membrane proteins and receptors, have been documented. Kagan and colleagues [59] demonstrated that LOX oxidizes specific cell surface proteins, such as the Platelet Derived Growth Factor Receptor-β (PDGFR-β) (Figure 2), to affect PDGF-BB-induced chemotaxis of aortic SMCs by enhancing phosphorylation of the PDGFR-β-dependent signal transduction pathway. Taken together, it is possible that lysyl oxidases have specific autonomous intracellular activities that are independent of their canonical role in ECM remodeling or other signal transduction pathways at the cells’ exterior and that both pathways can also act in concert to fine tune the ECM–cell interaction. 

It is intriguing to understand how an extracellular enzyme is found within the cell and, moreover, within the nucleus. There is no evidence of receptor uptake of any lysyl oxidases into the cell or for a classical nuclear localization signal (NLS) except for one in LOXL3 N-terminus [60]. Mutations in a possible NLS domain of LOX (similar to the NLS identified in N-myc) did not interfere with the nuclear localization of the enzyme, raising the possibility that this localization is mediated via a chaperon [41,59]. Nellaiappen raised a possible pathway by which LOX can enter the nucleus. In their work, SMCs incubated with a fluorescent or isotopically labeled LOX were shown to contain cytoplasmic and nuclear LOX and have raised the possibility that, once pro-LOX is secreted to the ECM and undergoes a proteolytic cleavage to yield the mature enzyme, it can subsequently re-enter the cell and concentrate within the nucleus [61]. An alternative mechanism for regulating intracellular localization of a LOX family member highlighted the role of the SRCR repeats. In their work, Ma et al. identified a putative nuclear export signal (NES) motif in LOXL3 N-terminus [38], whereas the SRCR repeats of the protein regulate its nuclear localization (Figure 1). Accordingly, LOXL3-deleted forms, which lack its C-terminus and the N-terminal NES localize to the nucleus, suggesting the SRCR regulates the subcellular localization [38]. These hypotheses are not complete as they cannot explain findings from various studies including ours, which demonstrate intrinsic and autonomous roles for lysyl oxidases. It is thus possible that additional mechanisms regulate lysyl oxidase uptake, cellular retention, and activity. Further studies should be carried out to elucidate this phenomenon.

Altogether, much work is still required to understand what regulates lysyl oxidase subcellular localization. The balance between their intracellular and extracellular localization plays a central role in their underlying activities, throughout development, and in pathological situations. Hence, mechanisms that regulate the export versus cellular retention or import will be a major focus for future research. Recent work aiming to identify novel partners for the LOX-family members could serve as an entry point into addressing this question [62]. The ability to control their localization could thus serve as a tool to direct the activities of this potent, multifunctional family of enzymes towards cell differentiation, migration, or inhibition of fibrosis.
